# How Progress Came in English Medicine

**Published:** 1906-07-14

**Authors:** 


### The Hospital

A JOURNAL OF

The Medical Sciences and Hospital Administration.

VOL. XL.—No. 1,034. SATURDAY, JULY 14, 1906.

### HOW PROGRESS CAME IN ENGLISH MEDICINE.

MEDICINE is undoubtedly progressive. Like all advances in the arts and sciences, its progress has been intermittent. The great periods of advance in English medicine have not necessarily or usually coincided with a renascence in other fields of thought or knowledge. During the Reformation our professional forefathers were struggling to secure a corporate existence, and cared but little for the science and practice of their art. Their efforts culminated on the physician's side in 1518, when letters patent were issued constituting the Royal College of Physicians of London, and in 1540, when the United Company of Barbers and Surgeons was established to give a recognised and definite professional status to the medical practitioners in London and its immediate neighbourhood. The spacious times of great Elizabeth left the surgeons the better for the contemporary literary activity. They learnt to write, to observe, and to compare. Clowes, Gale, Woodall, and Wiseman became the founders of modern surgery, which they raised from a subordinate place to a definite rank. But the physician remained a mere literary man, and was still to all intents and purposes a schoolman. The general practitioner had not yet come into existence, and his place was taken by the barber with some knowledge of surgery and a smattering of drugs. In 1617 these tradesmen had raised themselves, and in the most dead time of English history obtained incorporation and became apothecaries in the modern sense of the term. The apothecary soon reacted on the physician by demanding knowledge of him in difficult cases and by giving him opportunities of studying disease at the bedside. They compelled him to learn and to teach, though he still remained wedded to theory and a declared member of some sect in medicine. Again at a dead period in the history of England the surgeons divorced themselves from the barbers, and in 1745 entered upon a separate corporate existence. Each branch of the profession as it is at present constituted thus obtained distinct corporate existence—the physicians, the surgeons, and the apothecaries. As soon as this was accomplished the more active minds were able to devote themselves to advance their respective studies instead of concerning themselves with the improvement of their social status. The experimental methods of Harvey, learnt from the Italian School of Medicine, not only gave great results in themselves, but were important in the impetus they gave to the Oxford School of *virtuosi*, which, beginning with the more careful study of anatomy, ended in the foundation of the Royal Society. Neither surgeon nor apothecary had a share in this movement, which was limited to the physicians. The time of the surgeons did not come until the middle of the eighteenth century, when about 1740 Cheselden, Nourse, and Pott laid the foundations of modern surgery. Percivall Pott and William Hunter were the mainsprings of the revival of medical teaching, which is so great a feature of the early years of the reign of George III. They are responsible for the education of John Hunter, who raised the standard of surgical knowledge immeasurably, and himself founded the English School of Pathology which, in the hands of Matthew Baillie and his successors, became the present morbid anatomy. A body of well-educated, thoughtful, and observant men were sent out from Hunter's lecture theatre. The standard of professional knowledge increased steadily throughout the Hanoverian period, until the demand arose for a more thoroughly trained medical practitioner. This demand was met by the Apothecaries' Act of 1815, when the physicians had improved their clinical methods by the use of the stethoscope and by the study of morbid anatomy; the surgeons learnt anatomy with the utmost diligence, and the general practitioners had become expert classificatory botanists, a training most excellent, for it taught them to compare minute differences and to draw inferences. Improved and cheaper microscopes still further helped to train the physician, and made him more dependent on facts and less speculative. The introduction of anæsthesia radically altered the work of the surgeon, and gave him greater scope, whilst the general practitioner lost in money but gained in position when he was paid for his visits and not for the blue pills and black draughts which he supplied.

The introduction of bacteriology has revolutionised medicine in all its branches, but there is no sign that we have reached finality in any single direction. The physician has still much to learn in serum therapy; the surgeon does not yet know which of his cases merely recover and which are cured by the operations he has performed. Both have to learn from the general practitioner; he alone has the patient under observation for a sufficient length of time to see the final results obtained by the advice of the physician and the action of the surgeon. It would be well if the practitioner gave the results of his observations more often, for he is content usually to watch the results without comment, and thereby medical literature is poorer and a fountain of desirable medical knowledge lost to the profession. Our columns are open for the specific purpose of giving the practitioner the very opportunity which is denied him in other realms of medical press work, and in these columns his observations can be preserved, and thereby serve to increase the vastness of the progress which medical science is constantly making.

